# Giant osteochondral body in a popliteal cyst

**DOI:** 10.4103/0019-5413.45331

**Published:** 2009

**Authors:** MS Dhillon, Prabhudev Prasad, Akshay Goel, Abheek Kar

**Affiliations:** Department of Orthopaedic Surgery, Post Graduate Institute of Medical Education and Research, Chandigarh, India; 1 Department of Orthopaedic Surgery, Govt Medical College, Sector 32, Chandigarh, India; 2 Department of Orthopaedics, Apollo Hospitals, Colombo, Sri Lanka

**Keywords:** Giant osteochondral body, popliteal cyst

## Abstract

Popliteal cysts, although commonly seen, are rarely associated with motion restriction or calcification. Radiological features are of soft-tissue swelling, with occasional reports of calcifications or small osteochondral bodies inside the cysts. We report a giant osteochondral body in a popliteal cyst, with significant mechanical block to flexion. This type of mass has to be differentiated from synovial osteochondromatosis, calcifications in the cyst, extraosseous and intraarticular osteochondromas. Complete excision of the cyst resulted in complete recovery of range of motion.

## INTRODUCTION

Popliteal cysts are a common occurrence in orthopedic practice; many of these are innocuous, and are associated with degenerative arthritis or rheumatoid arthritis. Some may have loose calcified bodies in them. When the calcifications or bone formation are visible on standard x-rays, the differential diagnoses becomes more complex. The soft tissue tumors with calcification, such as liposarcoma or synovial sarcoma, are then also considered in difference diagnosis. Other reported causes range from popliteal artery aneurysms or arterovenous malformations, synovial chondromatosis, and post-traumatic loose bodies.^1^,^3^ A single giant osteochondral body in the popliteal cyst is rare, and to the best of our knowledge, only two such cases have been published in the English literature.^3^,^5^ No report however exists where the intraarticular findings of such a case were documented by either MRI or arthroscopy. We report one such case to highlight its rarity and the fact that flexion restriction occurred due to the large size of the osteochondral mass; the MRI findings, arthroscopic features, differential diagnosis, and management are discussed.

## CASE REPORT

A 53-year-old management executive with no comorbid medical illnesses presented with the complaint of gradually worsening pain in the right knee of 3 years duration. He was an active tennis player, and had to give up active sports due to pain and inability to fully bend his knee; he gradually developed a limp during walking. The patient had been vaguely diagnosed with gout 3 years before presentation, due to elevated serum uric acid and knee pain and had been taking allopurinol tablets orally since then.

Physical examination of the right knee revealed a flexion deformity of 5°, with further flexion up to 120° only. There was minimal effusion in the suprapateller pouch. McMurray's test was positive for medial joint pain and click was elicited. A nontender, firm to hard, fixed, nonpulsatile and well defined swelling was palpated in the popliteal fossa, medial to the medial head of gatrocnemius, which became prominent on full extension of the knee. Routine blood investigations, including the uric acid levels were unremarkable.

**Figure 1:**
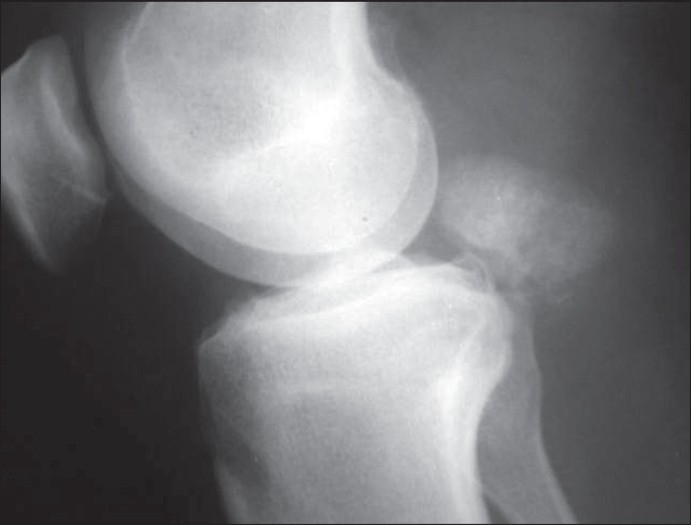
Lateral radiograph of the right knee showing a single calcified body

**Figure 2:**
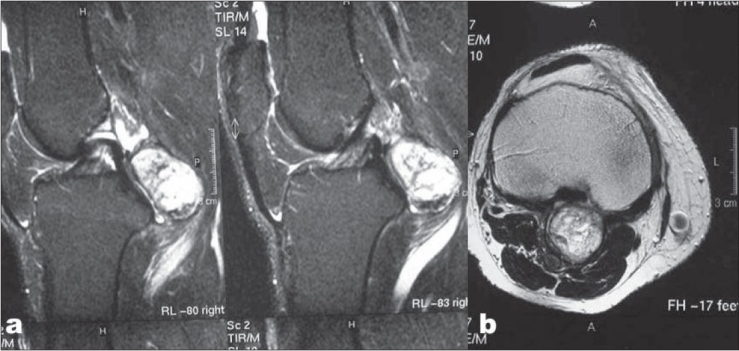
(a) Saggital and (b) axial MR images T2 W showing a hyperintense lesion in the popliteal fossa

Knee radiographs revealed a heterogeneously calcified, well defined mass measuring 3.8 × 3.2 cm in the popliteal fossa. Moderate degenerative changes in the medial compartment were also noted [Figure 1]. The left knee was essentially normal. An MR image of the right knee was subsequently obtained which revealed mild effusion in suprapateller pouch and radial tear of posterior horn of medial meniscus. On axial, sagittal, and coronal T2-weighted images [Figure 2a, Figure 2b], the mass in the popliteal fossa showed areas of increased signal intensity all over the lesion. There were no areas of signal void. The mass had displaced the popliteal neurovascular bundle posteriorly and was situated between the two heads of gastrocnemius.

**Figure 3:**
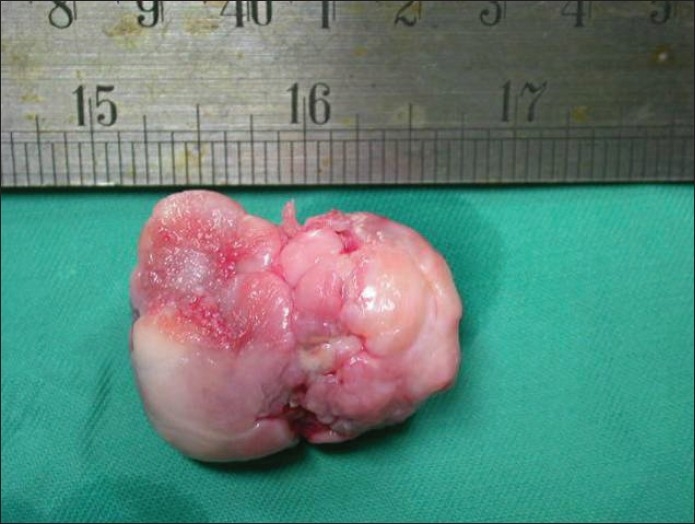
Gross morphology of the excised specimen showing cartilaginous cap

**Figure 4:**
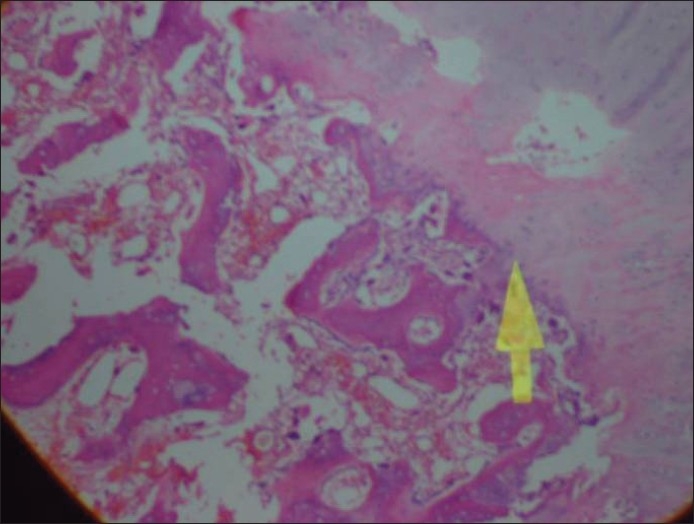
Histological picture showing cancellous bone in the center with a cartilaginous cap over it (×40)

An arthroscopy was planned to address the meniscal and intraarticular pathology; this revealed an undersurface tear of medial meniscus in the posterior horn, extending to the periphery. The inferior flap was excised, and a motorized chondroplasty was done for the worn out medial compartment of the knee. No obvious communication could be found to the popliteal fossa through the posterior capsule. The patient was then shifted into a semilateral position and the lesion was excised through a posteromedial longitudinal incision after retracting the medial head of gastrocnemius laterally. A well-encapsulated mass [Figure 3] was found adherent to the posterior capsule through a nonpatent narrow stalk. The capsule was opened, and a cartilage covered loose body was removed. The cyst wall and the stalk were also removed. Sections of the mass demonstrated a center of vascular cancellous bone surrounded by cartilage. Histopathological slides showed trabaculae of mature cancellous bone overlaid by cartilage all around, with some focal hyperplasia [Figure 4]. The synovium around the mass did not show any evidence of cartilaginous metaplasia. Findings were suggestive of an osteocartilaginous body with in a popliteal cyst. No evidence of gouty tophi was found. The patient had an uneventful recovery and regained a full range of movement in 6 weeks. At 1.5 years follow-up, he had restarted tennis (doubles) and had knee motion in range of 5–140°.

## DISCUSSION

A popliteal cyst was first described by Baker in 1877;^6^ this typically involves the gastrocnemius -semimembranosus bursa, which is located between the medial femoral condyle, semimembranosus tendon and the medial head of gastrocnemius.^7^ According to Meyerding and Van DeMark,^8^ these cysts are synovial lined and are usually associated with intraarticular pathology. Lesions such as meniscal tears or rheumatoid synovitis which result in chronic synovial effusion may be responsible for some popliteal cysts.^8^ In our case, we were able to demonstrate both on MRI and arthroscopy that in addition to knee joint degeneration, there was a medial meniscal tear in the posterior horn.

Radiographic evidence of calcification or ossification in the popliteal cyst is rare, and documented cases are mostly of multiple small loose bodies [Table 1].^1^,^2^,^3^,^9^ Single large ossified masses presenting as osteochondral lesions in the popliteal cyst have only been reported twice before.^3^,^5^ Smillie^5^ reported a popliteal cyst that contained a large osteochondral mass; he however considered it to be an example of synovial chondromatosis that was limited to popliteal cyst although there was no evidence of cartilage metaplasia in the cyst wall. Beneditti and coauthors^3^ reported on a case with a large double loose body, one of which was removed surgically. In none of the previously reported cases has there been any note of flexion restriction due to the large size of the mass, nor has there been documentation of the intraarticular pathology on arthroscopy. The detailed MRI findings have been presented for the first time in our case.

**Table 1:** Calcified bodies in a popliteal cyst reported in the literature

Author/ Year	No. of cases (Age/Sex)	Radiological findings	Histological diagnosis
Smillie^5^	1	Single calcified lesion	Osteocartilaginous body
Goldberg^2^	1 (64/F)	Multiple dense, round punctate calcifications	Cartilage growing from synovium with areas of calcification (synovial chondromatosis)
	2 (39/M)	Degenerative changes in knee with calcified bodies behind the knee and in the joint.	No cartilage metaplasia in synovium, loose bodies were cartilage fragments from femoral condyle
	3 (67/M)	Multiple calcified masses in the popliteal cyst.	Not done (radiological diagnosis of synovial chondromatosis)
	4 (70/M)	Multiple calcified masses in the popliteal cyst.	Not done (radiological diagnosis of synovial chondromatosis)
De Benedetti^3^	1 (61/ M)	A large ossific mass in the popliteal space and a smaller ossific mass at the level of the fibular head	A typical osteochondral loose body with well-defined zones of cartilage, subchondral bone, and cancellous bone. The cyst wall was smooth and contained no evidence of cartilaginous metaplasia
Kattapurum^1^	1 (61/F)	Spheroid, calcific mass in the popliteal space and vascular calcifications were also noted. The bony structures were unremarkable with no significant evidence of arthritis	A popliteal cyst with calcification in the fibrous layer of its wall.
Hertzanu *et al*,^9^	1 (62/F)	A large Baker's cyst with extension in to the calf with calcified opacities in the lower aspect	Not done (x-ray and arthrographic diagnosis of calcified bodies in the Baker's cyst)
Kullmer *et al*,^16^	1	Calcified concrements in the popliteal fossa after a femur fracture and a complex knee injury	Not done (radiological diagnosis of post-traumatic loose bodies in the joint)

Cases of synovial chondromatosis limited to the popliteal bursa have been reported.^10^–^12^ However, bursal synovial chondromatosis differs from the present case, as the cartilaginous or osteocartilaginous bodies would be multiple and smaller. Also, to establish a diagnosis of bursal synovial chondromatosis, it must be demonstrated that the cartilaginous or osteocartilaginous bodies had their origin in the bursal wall by cartilaginous metaplasia of the synovial connective tissue.^3^,^10^–^12^ In our case, the osteochondral body was lying loose inside the popliteal cyst; MRI was very helpful in evaluating the lesion, as a single discrete mass seemingly separate from the cyst wall was clearly visible.

The differential diagnosis may also include soft tissue tumors with calcification, such as liposarcoma or synovial sarcoma; internal calcification in synovial sarcoma, however, is usually peripheral and is less dense.^13^,^14^ A case of multiple small calcifications in a long-standing Baker cyst occurring in a patient with rheumatoid arthritis has also been reported. The authors postulated this was related to rapid joint destruction and multiple intraarticular and intracystic injections of triamcinolone.^15^ In some patients popliteal artery aneurysm can present as calcified popliteal mass; it is important to note that not all aneurysms will be pulsatile or have a bruit^3^. It is however, usually possible to differentiate an inhomogenously and less densely calcified aneurysm from a densely calcified osteochondromatous mass; the role of MRI comes into play in such situations, and the differentiation is quite easily done.

Kullmer *et al.*^16^ reported a case of popliteal cyst that contained calcified inclusions after a femur fracture and a complex knee injury. The etiology of the calcified bodies was thought to be due to post-traumatic loose bodies, rather than traumatically induced chondromatosis. Calcified bodies in a joint following trauma may be due to joint destruction or synovial osteochondromatosis. These loose bodies may pass in to a popliteal cyst through posterior joint bursal communications. Calcified loose bodies may also arise in a popliteal cyst de novo on account of chondrometaplasia.^16^

The unique nature of our case stems from the fact that the size of the osteochondral loose body had become quite large; restriction of flexion was probably due to mechanical restriction, as the range improved significantly after excision. The etiopathogenesis is a matter of conjecture, and we feel that the gradual onset of degeneration lead to fluid and debris accumulation in the popliteal cyst; this underwent some kind of metaplasia and developed into an osteochondral body. The patent communication with the joint ultimately closed down. Since gouty tophi have been implicated as a cause of deposition in the popliteal cyst, our first impression was that of gouty deposits. However, the MRI and histopathological findings both did not show any tophi.

Although arthroscopy did not help in the diagnosis of the current condition, its use lies in the fact that intraarticular lesions like meniscal tears can be addressed and communications of cysts into the joint can be localized. MRI gives excellent anatomic and three-dimensional localization and even demonstrated degeneration and meniscal tears, which we believe was part of the contributing pathology.

In summary, we report an unusual giant densely calcified osteochondromatous mass lying inside a popliteal cyst, which was responsible for mechanical loss of flexion. Such osteochondromatous lesions should be included in the differential diagnosis of all calcified masses in the popliteal space.
